# A case-report of widespread pulmonary embolism in a middle-aged male seven weeks after asymptomatic suspected COVID 19 infection

**DOI:** 10.1186/s12959-020-00235-w

**Published:** 2020-08-28

**Authors:** Mats Beckman, Sven Nyrén, Anna Kistner

**Affiliations:** 1 Department of Radiology, Imaging and Physiology, Karolinska University Hospital, Stockholm, Sweden; 2 Department of Molecular Medicine and Surgery, Karolinska Institutet, Stockholm, Sweden; 3 Medical Radiation Physics and Nuclear Medicine, Imaging and Physiology, Karolinska University Hospital, Stockholm, Sweden

**Keywords:** Pulmonary embolism, Covid-19, Male, Ground-glass

## Abstract

**Background:**

Pulmonary embolism (PE) is seen in high frequency in hospital-treated patients with Covid-19. We present a case of suspected Covid-19 with long-term dyspnea and widespread PE.

**Case presentation:**

A 51- year old male, with no prior medical history, no medication, and non-smoker arrived at the emergency department with exercise induced dyspnea during 4–5 weeks and for the last 48 h dyspnea at rest. Seven weeks before hospitalization, he felt difficulties taking deep breaths for some days but no other symptoms. Oxygen saturation at rest was 93%. Troponin T was 1200 mg/L (ref < 15 mg/L). CT angiography revealed widespread bilateral segmental pulmonary embolism. Additional findings were ground glass opacities that could match Covid-19. The patient tested negative for SARS -CoV-2. Full dose tinzaparin was given for 2 days in hospital, followed by apixaban for 6 months. Recovery has been uneventful so far.

**Conclusions:**

Long-term breathing difficulties might be relatively common after non-hospitalized symptomatic Covid-19. The frequency of PE in this group is unknown. We report a case of suspected covid-19 with widespread PE and a long history of dyspnea but no other symptoms. In our case slight hypoxia and laboratory testing indicated significant disease, which was proven with contrast angiography. This case shows that PE is a differential diagnosis in non-hospitalized symptomatic Covid-19 with persisting breathing problems.

## Background

Pulmonary embolism (PE) has been shown to be common in hospitalized Covid-19 patients with a 30% incidence [1]. In Sweden, infected subjects treated at home were not tested. Falling ill with fever and cough were regarded as typical Covid-19 infection and the recommendations from the Public Health Authority (FHM) in Sweden was “stay at home until you feel healthy and 48 hours thereafter” [2]. We report a case of suspected Covid-19 infection with widespread pulmonary embolism in a patient with no previous symptoms and severe pulmonary embolism.

## Case presentation

A 51- year old male, with no prior medical history, no medication, non-smoker and without risk factors for venous thrombo-embolism arrived at the end of April to the emergency department with exercise induced dyspnea during 4–5 weeks and for the last 48 h dyspnea also at rest. Prior to the onset of symptoms, he had lived socially isolated with his wife from mid-March, approximately 40 days, both working from home and with their two children home from school. He described a short period in the beginning of March, 7 weeks before hospitalization, when he felt difficulties taking deep breaths for a couple of days but no other symptoms like cough, fever or feeling of malaise. Following that episode he experienced a gradually increased fatigue on his regular run and in the beginning of April he had to start walking when running uphill. During the last 5 weeks before hospitalization his wife and daughter had noticed signs of heavy breathing when he walked up the stairs.

**Fig. 1:**
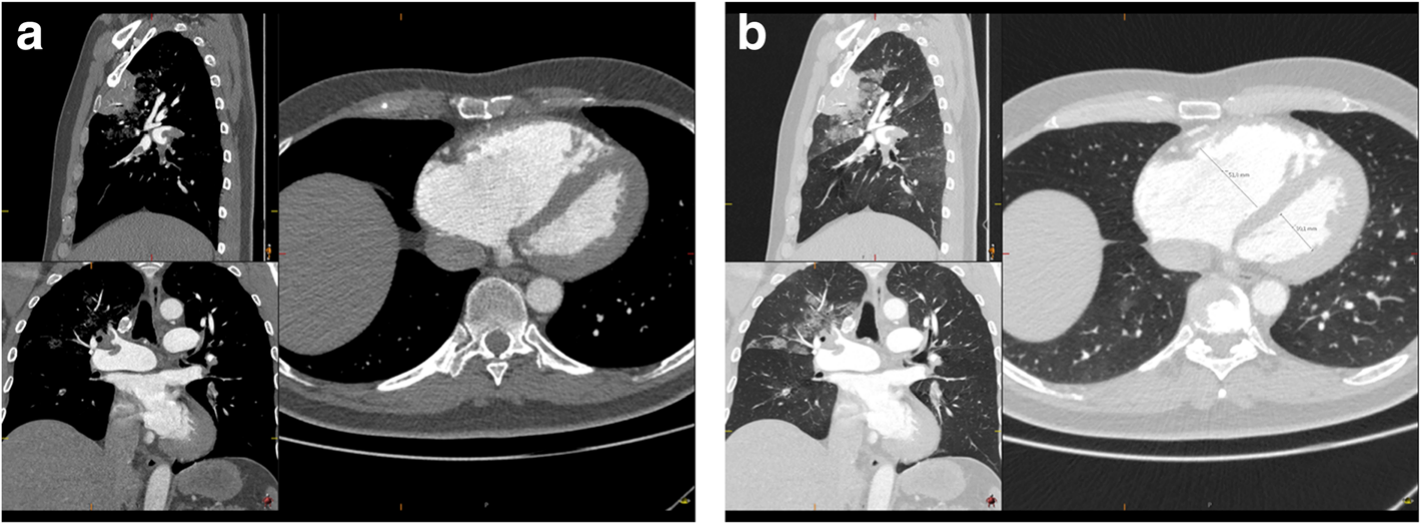
**a** and **b** Widespread bilateral pulmonary embolism with right ventricular affection and a right ventricular to left ventricular quotient of 1,7 (ref < 0,9), as well as consolidation in the ventral part of the right upper lobe consistent with a suspected infarction

**Fig. 2:**
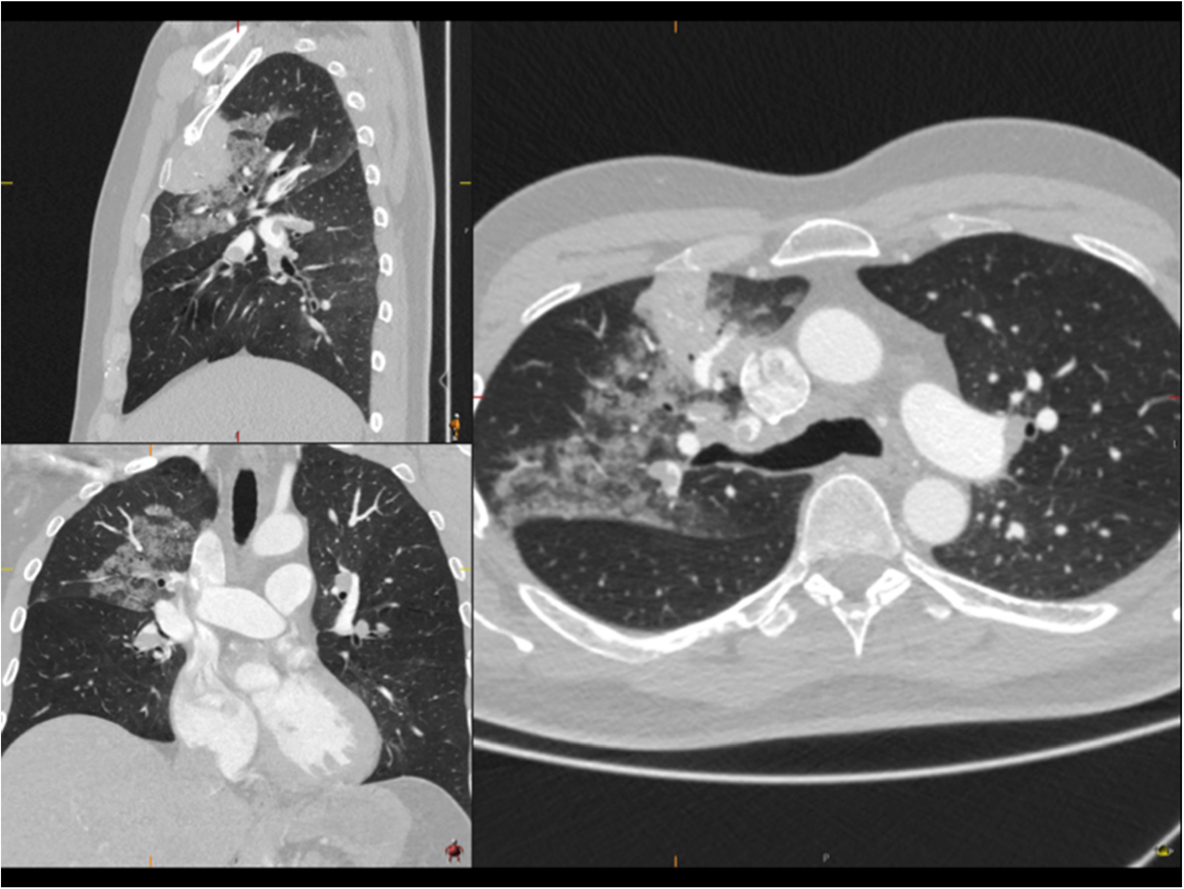
Ground glass opacities as well as the area of infarction in the right upper lobe

Physical examination was normal, examination of the heart and lungs revealed no discrepancies, no swollen legs or other signs of cardiac decompensation. The bodyweight of the patient was 90 kg and his height was 1.88 m, body mass index (BMI) was 25,5 kg/m^2^. He had normal temperature and a regular heart rate of 80 beats/min**.** He had a blood pressure of 180/65 mmHg and an oxygen saturation of 93% breathing ambient air. High sensitivity Troponin T was markedly elevated, 1200 (reference < 15 mg/L) and also B-type natriuretic peptide was increased, 737 (reference < 125 ng/L). He had a slightly increased C-reactive protein of 15 (reference < 5 mg/L) and modest leukocytosis 11,7 (normal range 3,5–8,8 × 10^9^/L). ECG showed incomplete right-sided branch block. Computerized Tomography Angiography (CTA) of the chest was performed as pulmonary embolism was suspected. The CTA revealed widespread bilateral segmental pulmonary embolism (Fig. 1) and an additional area of consolidation in the right upper lobe consistent with infarction. Additional findings of ground glass opacities that could match Covid-19 were also found (Fig. 2). The patient tested negative for SARS -CoV-2 (polymerase chain reaction SARS -CoV-2, GeneXpert, Cepheid, Sunnyvale, CA, United States) at two consecutive nasopharynx tests. No antibody test was performed. The patient was given oxygen and subcutaneous low molecular weight heparin (LWMH), tinzaparin 18,000 units daily during 2 days of hospitalization and was discharged with apixaban 5 mg, twice daily, with a treatment recommendation for 6 months.

Echocardiography revealed dilated right chamber, midventricular diameter of 5 cm and left septum deviation, light to moderate insufficiency of the tricuspid valve with a velocity max of 4,2 m/s. Vena cava inferior showed normal width and breathing variation. Severe pulmonary hypertension with a systolic pulmonary pressure of approximately 75–80 mmHg (normal upper limit 35 mmHg) was present. No significant amount of pericardial fluid was present.

## Discussion and conclusions

This case of suspected asymptomatic Covid-19 infection with widespread pulmonary embolism 7 weeks after possible infection proves the complex nature of this disease. It indicates the importance of informing individuals with or without a previously suspected Covid-19 to be aware of the risk for complications during a long time period. It is of importance that subjects seek care if suffering dyspnea or swollen legs. Healthcare workers need to be informed about pulmonary embolism as a possible late complication in subjects not severely affected by the disease. A weakness of this study is that we do not have the definite diagnosis. However, nasopharynx-and serology tests are seldom performed on individuals without clinical symptoms. A negative virus test 7 weeks after a possible infection is to be anticipated. A commercial antibody test (Abbott Architect SARS-CoV-2 IgG, North Chicago, Illinois, United States) taken 10 weeks after hospital discharge was negative. T cell immunity, that has been shown to be robust in convalescent individuals with asymptomatic or mild Covid-19 [3], was not investigated.

From the combination of very light respiratory symptoms 7 weeks before the examination and Covid-19 typical consolidations on the CTA we find it very probable that the patient had a Covid-19 infection almost 2 months before the acute illness and a widespread pulmonary embolism.

Longitudinal studies in medically ill patients have shown that the majority of venous thrombosis events occur in the posthospital setting within 6 weeks of hospitalization [4]. Consensus is emerging and recommendations at the moment say that hospitalized patients with Covid-19 should receive anticoagulants. The present practice guidelines recommend thromboprophylaxis with subcutaneous LMWH twice daily at prophylactic or intermediate doses, to reduce thrombotic risk [5, 6]. Security considerations are important with dose reduction in renal insufficiency etc. Patients hospitalized with severe Covid-19 pneumonia, especially if obese (BMI > 30 kg/m^2^), might be at further increased risk for thombosis and now often receive full dose (therapeutic) anticoagulation from hospital admission [7]. Thus treatment and recommended doses has changed over time.

After hospital discharge from Covid-19, extended prophylaxis with LMWH or novel oral anticoagulants (NOAC) can reduce the risk of venous thrombosis event [8] and treatment with NOAC during 2 to 4 weeks after hospital discharge is common practice according to region Stockholm expert committee guidelines (janusinfo.se), sometimes for longer period. If venous thromboembolism has been detected during hospitalization, a treatment period of 3 to 6 months is recommended. The possible value of anticoagulants to non-hospitalized patients with Covid-19 is subject to investigation. The case discussed in this paper indicates a possible value of such antithrombotic treatment. It also shows that PE could be a differential diagnosis in non-hospitalized symptomatic Covid-19 with persisting breathing difficulties.

## Data Availability

Not applicable.

## Abbreviations

PE: Pulmonary embolism
FMH: Public Health Authority
CTA: Computerized Tomography Angiography
BMI: Body mass index
LWMH: Low molecular weight heparin
NOAC: Novel oral anticoagulants
